# Acute Kidney Injury in Acute Heart Failure Revisited: Marker of Cardiorenal Disease Severity Rather Than Isolated Renal Injury

**DOI:** 10.3390/life16030486

**Published:** 2026-03-17

**Authors:** Georgios Aletras, Maria Bachlitzanaki, Maria Stratinaki, Ioannis Petrakis, Yannis Pantazis, Emmanuel Foukarakis, Michalis Hamilos, Kostas Stylianou

**Affiliations:** 1 Department of Cardiology, Venizelio General Hospital of Heraklion, 71409 Heraklion, Greece; medp2012222@med.uoc.gr (G.A.); maria.stratinaki@gmail.com (M.S.); mfouk@hotmail.com (E.F.); 2 School of Medicine, University of Crete, 70013 Heraklion, Greece; petrakgia@uoc.gr (I.P.); mchamilos@uoc.gr (M.H.); 3 Second Department of Internal Medicine, Venizelio General Hospital of Heraklion, 71409 Heraklion, Greece; mariabachlitzanaki@gmail.com; 4 Department of Nephrology, University General Hospital of Heraklion, 71500 Heraklion, Greece; 5 Institution of Applied and Computational Mathematics, Foundation of Research and Technology-Hellas, 70013 Heraklion, Greece; pantazis@iacm.forth.gr; 6 Department of Cardiology, University General Hospital of Heraklion, 71500 Heraklion, Greece

**Keywords:** acute heart failure, acute kidney injury, renal function, cardiorenal continuum, congestion, biomarkers

## Abstract

**Background:** Renal function deterioration during hospitalization for acute heart failure (AHF) is common and is traditionally classified as acute kidney injury (AKI) or worsening renal function (WRF) based on changes in serum creatinine (Cr). However, Cr-based definitions may inadequately reflect the complex cardiorenal interactions occurring in AHF. **Purpose:** This narrative review summarizes and compares definitions of AKI and WRF used in AHF, evaluates their prognostic significance, and explores whether renal function deterioration should be interpreted as a marker of cardiorenal disease severity rather than isolated kidney injury. **Methods:** A narrative review of randomized trials, observational studies, post hoc analyses, and meta-analyses was conducted, focusing on Cr-based and nephrology-derived AKI definitions (RIFLE, AKIN, KDIGO), timing and baseline selection, congestion status, and the role of biomarkers and imaging in clinical interpretation. **Results:** The most widely used definition of WRF is an absolute increase in serum Cr ≥ 0.3 mg/dL. Multiple studies demonstrate that such changes frequently occur during effective decongestion and are not independently associated with adverse outcomes in the absence of residual congestion. In contrast, persistent congestion, impaired diuretic response, reduced renal reserve, and advanced cardiorenal comorbidity consistently predict worse prognosis. Nephrology-derived AKI definitions identify higher-risk patients but incompletely account for the hemodynamic and therapeutic context of AHF. **Conclusions:** In AHF, AKI and WRF often act as markers of underlying cardiorenal disease severity rather than direct indicators of irreversible kidney injury. Interpretation of renal function deterioration should be contextual, integrating congestion status, perfusion, renal reserve, and dynamic response to therapy. Achieving effective and complete decongestion remains the primary therapeutic objective in AHF, even in the presence of transient Cr increases.

## 1. Introduction

Acute heart failure (AHF) remains one of the leading causes of hospitalization among individuals aged over 65 years and is associated with substantial short- and long-term mortality, as well as high rates of rehospitalization [1]. Contemporary management focuses on rapid hemodynamic stabilization, effective relief of congestion, and early initiation or optimization of guideline-directed medical therapy (GDMT) [2]. However, during decongestive treatment and GDMT implementation, clinicians are frequently confronted with a decline in kidney function, most commonly assessed by changes in serum creatinine (Cr) and Cr-based estimated glomerular filtration rate (eGFR). This phenomenon, often labeled as worsening renal function (WRF) or acute kidney injury (AKI), occurs in approximately 30–50% of patients hospitalized with AHF, with even higher rates reported in selected cohorts [3,4,5].

The interpretation and management of renal function deterioration during AHF hospitalization remain challenging. A central clinical dilemma lies in distinguishing true WRF—reflecting structural kidney injury—from functional, hemodynamically mediated increases in serum Cr that occur during aggressive decongestion or neurohormonal modulation. Despite growing evidence that such functional changes may be transient or even prognostically neutral, misinterpretation of Cr rises continues to lead to inappropriate reduction or discontinuation of diuretic therapy and disease-modifying agents in everyday practice. Paradoxically, patients with pre-existing chronic kidney disease (CKD), who are at the highest risk of WRF, may derive the greatest absolute benefit from neurohormonal blockade owing to their elevated baseline cardiovascular risk [3,6].

In the AHF literature, the terms WRF and AKI are frequently used interchangeably, despite important conceptual differences. In parallel, nephrology has introduced several creatinine-based classification systems for AKI, including RIFLE, AKIN, and KDIGO criteria (Table 1). However, these definitions were largely developed in non-HF populations and vary substantially with respect to absolute versus relative Cr thresholds, timing of assessment, and choice of renal marker (serum Cr, eGFR, or alternative biomarkers such as cystatin C). Consequently, their applicability and prognostic relevance in the setting of AHF remain uncertain [7,8,9].

AHF represents a unique pathophysiological state in which renal function is influenced by multiple interacting mechanisms, including venous congestion, altered renal perfusion, neurohormonal activation, and the effects of pharmacological therapies. Within this context, Cr-based definitions may inadequately capture the complexity of renal function changes [8,10]. Indeed, increases in serum Cr during decongestive therapy often prompt clinicians to reduce diuretic intensity under the assumption of impending tubular injury, despite evidence that persistent congestion at discharge is associated with markedly worse outcomes—even in the presence of WRF. Conversely, an apparent improvement in Cr may offer false reassurance, masking ongoing congestion and suboptimal volume status [3,11,12].

Similar interpretive challenges arise during up-titration of neurohormonal therapies, where modest Cr increases frequently limit treatment optimization, despite their proven prognostic benefit. Although contextual interpretation of renal function deterioration has been increasingly acknowledged in recent clinical guidelines, substantial heterogeneity persists across studies regarding definitions, baseline Cr selection, timing of assessment, and integration of congestion status [6,13,14].

Against this background, the present narrative review aims to reconcile these inconsistencies by framing AKI and WRF within a dynamic renal function trajectory model and by proposing a structured, multidomain approach to clinical interpretation in patients hospitalized with AHF.

## 2. Objectives

The objectives of this narrative review are:

1. To summarize and compare the definitions of AKI and WRF used in studies of AHF.
2. To analyze the sources of heterogeneity in reported prognostic associations, including differences in baseline Cr selection, timing of assessment, and clinical context.
3. To evaluate whether current creatinine-based definitions adequately reflect true kidney injury in the setting of AHF.
4. To conceptualize renal function deterioration as part of a dynamic renal trajectory during AHF hospitalization rather than a fixed categorical event.
5. To propose a structured, multidomain framework for contextual interpretation and clinical decision-making.

## 3. Literature Review Strategy

This narrative review was conducted using a structured, non-systematic literature search designed to identify studies addressing definitions, mechanisms, and prognostic implications of AKI and WRF in the setting of AHF.

The primary search was performed in PubMed/MEDLINE. To enhance completeness, supplementary searches were conducted using Scopus and Google Scholar, and reference lists of key reviews, randomized trials, and position statements were manually screened. The search included studies published up to February 2026. No strict lower time limit was imposed; however, emphasis was placed on studies published after the introduction of standardized AKI definitions (post-RIFLE era, 2004 onward).

The search strategy combined Medical Subject Headings (MeSH) and free-text terms using Boolean operators. Core search strings included combinations of:

▪ “acute heart failure” OR “acute decompensated heart failure”
▪ AND “acute kidney injury” OR “worsening renal function”
▪ AND “cardiorenal syndrome” OR “type 1 cardiorenal syndrome”
▪ AND “creatinine” OR “cystatin” OR “renal biomarkers”
▪ AND “congestion” OR “decongestion” OR “renal reserve”

Searches were iteratively refined to identify studies specifically addressing:

1. Creatinine-based AKI definitions or WRF definitions in AHF,
2. Nephrology-derived classifications (RIFLE, AKIN, KDIGO),
3. Baseline creatinine selection and timing of renal function assessment,
4. Congestion status and hemodynamic modifiers
5. Biomarkers of renal filtration, tubular injury or cellular stress.

Eligible studies included randomized controlled trials (RCT), prospective and retrospective observational cohorts, post hoc analyses of clinical trials, systematic reviews, and metanalyses published in English. Studies were required to report renal function changes in hospitalized AHF populations and examine clinical outcomes. Case reports and small case series were excluded, unless they provided mechanistic insights relevant to cardiorenal pathophysiology.

Studies focusing on cardiogenic shock or profound hemodynamic instability were not the central focus of this review, given the distinct mechanisms of AKI in those settings; such studies were considered separately when relevant to mechanistic interpretation.

Given the narrative design, no formal quantitative quality scoring tool was applied. However, study selection for detailed discussion and inclusion in the summary table was guided by predefined criteria, including:

1. Clarity of AKI/WRF definition
2. Specification of baseline creatinine
3. Timing of renal function assessment
4. Adjustment for confounders in outcome analyses, and
5. Clinical relevance to congestion-predominant AHF

When multiple analyses derived from the same parent cohort were identified, the most methodologically comprehensive or clinically informative report was prioritized to minimize duplication, while additional analyses were included only when addressing distinct mechanistic or prognostic questions. The evidence was synthesized qualitatively and organized thematically according to definitional frameworks, renal function trajectories, congestion status, baseline renal vulnerability, and biomarker integration.

## 4. Pathophysiological Background

Renal dysfunction in AHF is the result of a complex and dynamic interplay among hemodynamic alterations, neurohormonal activation, inflammatory pathways, and treatment-related effects. This multifactorial process underpins acute cardiorenal syndrome (ACRS) and accounts for the frequent difficulty in interpreting changes in renal function during hospitalization [4,15,16].

Historically, WRF in AHF was primarily attributed to reduced cardiac output and impaired renal perfusion. Although low cardiac output can contribute to renal dysfunction in certain patients, particularly those with advanced HF or cardiogenic shock, it is not the predominant mechanism in most cases of acute decompensation. Multiple studies have demonstrated a weak or inconsistent association between cardiac output and changes in renal function in AHF, indicating that other mechanisms are more influential [6,12,17].

There is growing evidence that elevated central venous pressure (CVP) and renal venous congestion are primary contributors to renal dysfunction in AHF. Volume overload increases right-sided filling pressures, raises intra-abdominal pressure, and impairs renal venous drainage. These alterations decrease the transglomerular pressure gradient, slow intrarenal blood flow, and impair glomerular filtration, leading to reduced urine output and increased serum Cr. Notably, venous congestion directly impairs renal function independently of arterial perfusion, which explains renal deterioration in patients with preserved or only mildly reduced cardiac output [3,10,17,18].

In addition to venous congestion, arterial pulsatile hemodynamics may also contribute to cardiorenal interactions in AHF. Increased arterial stiffness, widened pulse pressure, and enhanced pulsatile load can transmit excessive systolic energy into the renal microcirculation, promoting glomerular injury and impaired autoregulation. In patients with long-standing hypertension, diabetes, or vascular aging, reduced arterial compliance may amplify renal vulnerability to hemodynamic stress during acute decompensation. Recent work has highlighted the role of pulsatile arterial load and wave reflection in modulating renal perfusion and risk stratification in cardiovascular disease, suggesting that adverse arterial hemodynamics may act synergistically with venous congestion in promoting renal dysfunction [19,20,21].

Beyond these hemodynamic determinants, neurohormonal activation represents another central mechanism in acute cardiorenal interactions. Activation of the renin–angiotensin–aldosterone system (RAAS) and the sympathetic nervous system serves as a compensatory mechanism to maintain systemic perfusion and arterial pressure. However, persistent activation of these pathways induces renal vasoconstriction, sodium and water retention, and maladaptive intrarenal hemodynamic changes. Angiotensin II preferentially constricts the efferent arteriole, modifies intraglomerular pressure, increases proximal tubular sodium reabsorption, and elevates peritubular oncotic pressure, thereby reducing glomerular filtration. Concurrently, angiotensin II stimulates endothelin-1 production, and aldosterone increases distal tubular sodium reabsorption, together promoting inflammation, fibrosis, and microvascular dysfunction in the kidney [15,17,22].

Inflammation and oxidative stress further increase renal vulnerability in AHF. Neurohormonal overactivation, venous congestion, and tissue hypoxia stimulate the release of pro-inflammatory cytokines and reactive oxygen species, which impair endothelial function and worsen tubular injury. Elevated oxidative stress markers have been observed in patients with acute decompensated heart failure (ADHF) and ACRS, supporting the significant contribution of non-hemodynamic mechanisms to renal dysfunction in this context [23].

Treatment-related factors further complicate the clinical picture. Aggressive decongestive therapy often leads to transient increases in serum Cr, which may indicate hemoconcentration, changes in intraglomerular pressure, or restoration of baseline renal function after relief of congestion, rather than structural kidney injury. Similarly, initiation or up-titration of renin–angiotensin–aldosterone system inhibitors (RAASi) and sodium–glucose cotransporter-2 inhibitors (SGLT2i) is frequently associated with modest early increases in serum Cr. These changes are primarily due to predictable alterations in afferent and efferent arteriolar tone and intraglomerular hemodynamics, and they are recognized as precursors to long-term renal protection and slower progression of CKD [24,25,26].

Importantly, serum Cr itself is an imperfect indicator of renal injury in AHF. In patients with congestion, Cr concentrations may be falsely low due to hemodilution, resulting in overestimation of glomerular filtration. During decongestion, rising Cr may reflect normalization toward baseline values rather than new kidney injury. Therefore, knowledge of pre-admission renal function and longitudinal Cr trends is essential for accurate interpretation [3,27,28].

Taken together, these mechanisms demonstrate that renal function deterioration in AHF represents a spectrum, ranging from transient functional changes to true structural kidney injury. The primary clinical challenge is to distinguish between these entities, as their prognostic significance and therapeutic implications differ considerably [3,6,17].

## 5. Definitions of AKI and WRF in Acute HF

Renal function deterioration during hospitalization for AHF has been described using a wide range of definitions, contributing substantially to heterogeneity in reported incidence and prognostic associations. In the cardiology literature, the most commonly applied concept is WRF, typically defined as an absolute increase in serum Cr of ≥0.3 mg/dL during hospitalization, sometimes complemented by relative changes from baseline. This threshold has been adopted largely for its simplicity and reproducibility and remains the most frequently used definition across observational cohorts, randomized trials, and post hoc analyses [27,29,30,31].

Despite its widespread use, this Cr-based definition captures a biologically heterogeneous group of patients. Several studies have shown that an isolated rise in serum Cr does not uniformly reflect structural kidney injury, particularly in the context of aggressive decongestive therapy. Randomized and observational data consistently indicate that WRF defined by a ≥0.3 mg/dL Cr increase may occur as a consequence of effective diuresis, hemoconcentration, or predictable intraglomerular hemodynamic changes, rather than intrinsic renal damage. Accordingly, multiple studies indicate that the prognostic significance of WRF is strongly influenced by the accompanying clinical context [3,27,30,32].

To address this limitation, these studies incorporated markers of congestion and demonstrated that the prognostic significance of Cr-based WRF depends strongly on residual congestion, which consistently emerges as a major determinant of risk. These findings challenge the interpretation of Cr changes in isolation and suggest that congestion status functions as a key modifier rather than an alternative definition [27,30,32,33].

Nephrology-derived definitions of AKI, including RIFLE, AKIN, and KDIGO criteria, have also been applied to AHF populations. These frameworks emphasize standardized staging based on absolute or relative Cr increases and, in some cases, urine output. While such definitions improve consistency across studies, their prognostic performance in AHF has been variable. Meta-analytic data confirm that AKI defined by these criteria is common in AHF and is associated with increased mortality and longer hospitalization, particularly with increasing severity or need for renal replacement therapy. However, these definitions were developed in settings such as sepsis, surgery, or critical illness and do not fully account for the hemodynamic and therapeutic context of AHF [29].

Beyond the magnitude of renal function change, several studies highlight the importance of timing in defining AKI and WRF. Early versus late onset of renal deterioration during hospitalization appears to identify distinct clinical phenotypes; however, evidence regarding the prognostic significance of early versus late AKI or WRF is conflicting across studies, suggesting that timing alone may not be sufficient to characterize clinically meaningful renal injury in AHF [34,35,36,37]. Similarly, the choice of baseline renal function—whether based on pre-hospital outpatient values or admission Cr—significantly affects AKI detection rates, risk stratification and observed prognostic associations. A major source of heterogeneity across studies relates directly to this methodological difference. When admission Cr is treated as the reference baseline, community-acquired AKI present at hospital arrival may be misclassified as non-AKI. Such misclassification may underestimate AKI incidence and attenuate the true strength of its association with adverse outcomes. In contrast, studies using pre-admission renal function as reference may better capture dynamic renal deterioration preceding hospitalization and often report higher AKI prevalence together with stronger and more consistent prognostic associations. However, this strategy is not without limitations. When baseline Cr values are obtained months prior to admission, rapidly progressive CKD may be misclassified as AKI, particularly in patients with accelerated decline in renal function. Thus, both admission-based and pre-admission baseline strategies carry inherent methodological constraints. Admission-based definitions may underestimate community-acquired AKI, whereas pre-admission reference values may overestimate AKI by misclassifying rapidly progressive CKD as acute injury. For this reason, neither strategy can be categorically excluded. Rather, inclusion of both approaches across studies may, to some extent, counterbalance their respective biases. Baseline selection, therefore, represents a methodological trade-off rather than a universally superior approach, and this variability likely contributes to discordant findings across cohorts [38,39,40].

Taken together, these definitional differences suggest that Cr-based AKI and WRF categories may represent different time points along evolving renal function trajectories during AHF hospitalization rather than strictly discrete events. Across studies, the prognostic meaning of renal deterioration depends not only on the magnitude of Cr change but also on its temporal pattern, baseline reference, and clinical context. Although the classic definition of WRF (ΔSCr ≥0.3 mg/dL) remains practical and widely used, its clinical and prognostic interpretation is inherently context-dependent. Definitions incorporating congestion status, timing of onset, baseline renal reserve, and persistence of dysfunction appear more closely aligned with clinically meaningful kidney injury and help explain variability in reported outcomes across studies (Table 2) [30,32,33,34,41].

**Table 2:** Representative studies evaluating definitions, modifiers, and prognostic implications of AKI and worsening renal function in acute heart failure. This table summarizes key randomized trials, observational cohorts, and meta-analyses that have evaluated acute kidney injury (AKI) and worsening renal function (WRF) in patients hospitalized for acute heart failure. Studies are grouped according to the definition used, associated risk factors or clinical modifiers and their reported prognostic implications. Collectively, these studies illustrate that the prognostic significance of creatinine-based renal function deterioration varies substantially according to clinical context, baseline definition, timing of assessment, and accompanying markers of congestion or hemodynamic severity. Studies are presented using their original AKI or WRF definitions, including baseline reference and time window of evaluation; however, findings are interpreted within the broader framework of dynamic renal function trajectories during AHF hospitalization. Abbreviations: ACS, acute coronary syndrome; ACE, angiotensin converting enzyme; AHF, acute heart failure; AKI, acute kidney injury; BNP, B-type natriuretic peptide; CKD, chronic kidney disease; CRS-1, cardiorenal syndrome type 1; CVP, central venous pressure; eGFR, estimated glomerular filtration rate; Hb, hemoglobin; Hct, hematocrit; HF, heart failure; HFpEF, heart failure with preserved ejection fraction; ICU, intensive care unit; KDIGO, Kidney Disease: Improving Global Outcomes; NT-proBNP, N-terminal pro-B-type natriuretic peptide; PCWP, pulmonary capillary wedge pressure; RCT, randomized controlled trial; RIFLE, Risk–Injury–Failure–Loss–End-stage; RRT, renal replacement therapy; SCr, serum creatinine; WRF, worsening renal function.

Study & Reference	Study Type	AKI/WRF Definition	Risk Factors for AKI/WRF	Key Findings & Prognostic Insight
**DOSE-AHF (2011)** *N Engl J Med* [31]	Double -Blind RCT (*N* = 308)	WRF: ΔSCr ≥ 0.3 mg/dL within 72 h.	High-dose loop diuretics (2.5 × home oral dose)	High-dose diuretics caused more frequent WRF (23% vs. 14%, *p* = 0.04) but better symptom relief; WRF did not increase 60-day clinical events.
**Testani et al. (SOLVD investigators)** **(2011)** *Circ Heart Fail* [42]	Post hoc analysis of RCT (*N* = 6337)	Early WRF: ≥20% decrease in estimated GFR at 14 days after randomization	Definition-dependent susceptibility: preserved baseline renal function (lower baseline creatinine; greater relative decline detectable), exposure to ACE inhibitor therapy Modifier: mechanism of WRF (pharmacologically mediated vs. spontaneous)	Early WRF was associated with increased mortality in placebo-treated patients but not in patients receiving enalapril. In the ACE inhibitor group, early WRF had no adverse prognostic significance, and continuation of enalapril despite WRF was associated with a survival benefit. The **mechanism underlying WRF critically determines its prognostic meaning**, with pharmacologically mediated WRF representing a largely benign phenomenon.
**Metra et al. (2012)** *Circ Heart Fail* [33]	Prospective Observational (*N* = 594)	WRF: ΔSCr ≥ 0.3 mg/dL from admission values	Lower-risk phenotype without WRF or congestion → Younger age, Fewer comorbidities, Preserved LVEF, Better renal profile, Less intensive therapy	WRF alone was not independently associated with mortality or rehospitalization. Adverse outcomes were driven by persistent congestion, with WRF conferring additive prognostic risk only when congestion persisted.
**Vandenberghe (2016)** *Cardiorenal Med* [29]	Systematic Review & Meta-analysis (64 papers—*N* = 509,766)	CRS-1 defined by AKI (RIFLE, AKIN, KDIGO), WRF, or need for RRT	Predisposing condition: Acute heart failure (vs. ACS or cardiac surgery)	AKI was most frequent in AHF. AKI was associated with markedly increased mortality and longer ICU and hospital stay, with risk rising stepwise with AKI severity; RRT conferred the worst prognosis.
**Takaya et al. (2016)** *Heart and Vessels* [35]	Retrospective observational study (*N* = 371)	ΔSCr ≥ 0.3 mg/dL or ≥1.5-fold within 48 h	Late AKI onset (≥5 days) associated with higher admission BUN and intravenous dobutamine use	Timing of AKI onset was prognostically relevant: Late-onset AKI (≥5 days) → independently associated with higher 12-month mortality Early onset AKI → no significant association with adverse outcomes.
**ESCAPE trial (2018)** *Am Heart J* [12]	RCT Sub-analysis (*N *= 433)	WRF: ΔSCr ≥ 0.3 mg/dL during hospitalization	Modifiers: Hemodynamics (RAP/PCWP), Clinical signs, Hemoconcentration, Plasma volume	WRF was not associated with 180-day death if patients were decongested, regardless of the measure used. Poor agreement between hemodynamic vs. clinical decongestion (43% mismatch).
**Metra et al. (2018)** *Circ Heart Fail* [27]	Post Hoc analysis (*N *= 1684)	WRF: ΔSCr ≥ 0.3 mg/dL from baseline	Modifier: Congestion status at time of renal function assessment	WRF predicted longer hospital stay and worse short- and mid-term outcomes **only in the presence of residual congestion**; SCr rise had limited prognostic impact in non-congested patients.
**Sanchez-Serna et al. (2020)** *Eur J Intern Med* [38]	Single-center observational cohort (*N* = 458)	AKI defined by KDIGO criteria using two baselines: Pre-hospital outpatient SCr vs. First in-hospital SCr	Definition-dependent: Use of pre-hospital baseline kidney function	Using pre-hospital renal function nearly doubled AKI detection and identified AKI already present at admission. **Only AKI defined using pre-hospital baseline** was independently associated with post-discharge mortality and HF readmission.
**McCallum et al. (2020)** *JACC Heart Fail* [43]	Post hoc analysis of RCT (EVEREST) (*N* = 3715)	Acute decline in eGFR during hospitalization (analyzed as % decline)	Modifier: Degree of decongestion and hemoconcentration	Acute declines in eGFR were associated with higher mortality and HF hospitalization only in the absence of decongestion. No prognostic harm when congestion or hemoconcentration markers improved → decongestion is a key modifier of renal prognostic significance
**Yamada et al. (2020)** *Am J Cardiol* [32]	Systematic Review & Meta-analysis (13 studies, *N* = 8138)	WRF: Absolute ΔSCr ≥ 0.3 mg/dL or relative ΔSCr ≥ 20–25%	Modifier: Decongestion status (Physical signs, BNP reduction, increase in Hb/Hct)	WRF without decongestion is harmful (OR 1.71), but WRF with decongestion has a neutral prognosis (OR 1.15). WRF + decongestion is better than no WRF + persistent congestion (OR 0.63).
**Diebold et al. (2020)** *ESC Heart Fail* [34]	Prospective observational cohort (*N* = 1643)	AKI: KDIGO SCr criteria (≥0.3 mg/dL within 48 h or ≥1.5× baseline within 7 days). Classification as community-acquired (present at admission) vs. in-hospital AKI	CA-AKI associated with lower transglomerular pressure gradient and tubular injury (elevated urinary NGAL)	Community-acquired AKI, but not in-hospital AKI, was independently associated with increased long-term mortality. CA-AKI showed evidence of structural injury and impaired transglomerular pressure, whereas in-hospital AKI appeared predominantly hemodynamic. Adequate decongestion was associated with lower mortality irrespective of AKI timing.
**Emmens et al. (2022)** *Eur. J. Heart F* [30]	Post hoc analysis of PROTECT (*N* = 1698) and RELAX-AHF-2 (*N* = 5586) cohorts	WRF: ΔSCr ≥ 0.3 mg/dL from baseline to day 4	Lower baseline eGFR, higher LVEF, higher loop diuretic doses, and poorer diuretic response	WRF was associated with worse outcomes only in patients with poor diuretic response; no adverse prognostic impact when effective decongestion was achieved.
**Presume et al. (2023)** *Rev Port Cardiol* [36]	Retrospective observational cohort (*N* = 249)	WRF: ΔSCr ≥ 0.3 mg/dL during hospitalization; Early WRF ≤ 48 h, Late WRF > 48 h; AKI at admission: ΔSCr ≥ 0.3 mg/dL from outpatient baseline	Older age, higher baseline/admission SCr; Early WRF phenotype more frequent in HFpEF	Early WRF (≤48 h), but not late WRF, independently predicted adverse 1-year outcomes, highlighting timing as a key determinant of prognostic relevance.
**Zhao et al. (2023)** *Front. Cardiovasc. Med* [41]	Retrospective observational cohort (*N *= 440)	Changes in SCr during hospitalization Non-severe WRF: ΔSCr ≥0.3 to <0.5 mg/dL, Severe WRF: ΔSCr ≥0.5 mg/dL) Non-WRF ΔSCr <0.3 mg/dL	CKD, Hypertension, Higher admission potassium, Higher BUN/SCr/BNP; Greater loop diuretic use	Non-severe WRF was not associated with increased 1-year mortality, whereas severe WRF was. Low BNP at discharge mitigated the adverse prognostic effect of severe WRF, indicating that residual congestion strongly influences the clinical significance of Cr rise.
**Moises et al. (2025)** *Am J Kidney Dis* [37]	Retrospective observational study (*N* = 753)	Renal dysfunction assessed by baseline eGFR, in-hospital eGFR slope, and need for dialysis	Elevated baseline CVP and PCWP (markers of volume overload)	Higher right- and left-sided filling pressures were independently associated with lower baseline eGFR, steeper in-hospital eGFR decline, and increased long-term risk of dialysis. Short-term changes in CVP or PCWP were not associated with concomitant changes in eGFR → Dominant role of **baseline congestion severity**
**Aletras et al. (2025)** *J Clin Med* [44]	Prospective observational cohort (*N* = 218)	AKI: ΔSCr ≥0.3 mg/dL or ≥1.5× baseline	Older age, higher CKD stage, worse NYHA class, higher NT-proBNP, and lower hemoglobin. CKD stage and admission Cr change were independent predictors	AKI occurred in over half of hospitalized AHF patients. It was associated with higher in-hospital mortality, longer length of stay, greater need for vasoactive therapy, and increased 6-month readmission and mortality.

## 6. Biomarkers and Alternative Approaches

While Cr-based definitions remain central to the assessment of renal function deterioration in AHF, their limitations have prompted investigation into complementary biomarkers that may help distinguish functional changes from true kidney injury. These approaches do not replace traditional definitions of AKI or WRF, but rather provide additional pathophysiological and prognostic context that may improve clinical interpretation [3,33,45].

Cystatin C (Cys C) represents the most extensively studied alternative filtration marker in HF. Compared with serum Cr, cystatin C is less dependent on muscle mass and nutritional status and may therefore provide a more accurate estimate of glomerular filtration in frail, elderly, or cachectic patients [4,46,47]. In AHF populations, elevated Cys C levels at admission and discordance between creatinine- and Cys C–derived eGFR have been consistently associated with worse outcomes, suggesting that Cr alone may underestimate renal vulnerability in advanced disease states [48,49]. Nevertheless, Cys C remains influenced by non-GFR determinants, including inflammation, thyroid dysfunction, and corticosteroid use, and its role in defining AKI or WRF thresholds in AHF has not been standardized [3,47].

Beyond filtration markers, biomarkers of renal tubular injury offer insight into the underlying mechanism of renal function deterioration. Urinary or plasma neutrophil gelatinase–associated lipocalin (NGAL), kidney injury molecule-1 (KIM-1), and liver-type fatty acid–binding protein (L-FABP) have been evaluated as indicators of structural tubular damage. Importantly, mechanistic studies in AHF have shown that small-to-moderate deteriorations in renal function during aggressive decongestive therapy are not consistently accompanied by increases in these tubular injury markers, supporting the concept that many episodes of WRF reflect hemodynamic or functional changes rather than intrinsic kidney injury. Conversely, elevated baseline levels or rising trajectories during hospitalization may identify patients with pre-existing tubular vulnerability or evolving structural damage, warranting closer monitoring and reassessment of therapeutic strategy [3,50,51,52,53].

Albuminuria provides additional, albeit non-specific, information in this setting. In AHF, albuminuria may arise from glomerular injury, impaired tubular reabsorption, systemic endothelial dysfunction, or venous congestion. Changes in albuminuria during hospitalization, particularly improvement following decongestion, may support effective volume offloading and recovery of renal hemodynamics. However, its interpretation should remain contextual and integrated with other markers of congestion and renal function [54,55,56,57].

More recently, biomarkers of early tubular stress, such as the combination of tissue inhibitor of metalloproteinases-2 (TIMP-2) and insulin-like growth factor–binding protein 7 (IGFBP-7), have been proposed for early AKI risk stratification. These markers reflect cell-cycle arrest preceding overt tubular injury and are primarily validated in critically ill populations. Although their role in AHF is not yet well defined, elevated values may identify patients at increased risk of progression to clinically significant AKI and support intensified monitoring and nephroprotective measures [58,59].

**Figure 1:**
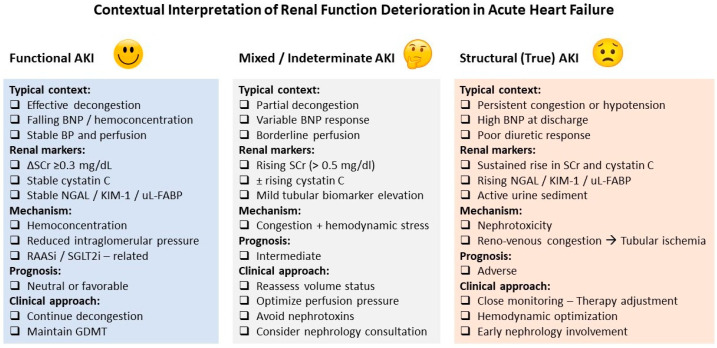
Contextual interpretation of renal function deterioration in acute heart failure. Conceptual framework illustrating three clinically relevant phenotypes of renal function deterioration in acute heart failure: functional AKI, mixed or indeterminate AKI, and structural (true) AKI. The classification integrates congestion status, renal biomarkers (serum creatinine, cystatin C, tubular injury markers), underlying mechanisms, prognostic implications, and corresponding clinical management strategies. This contextual approach highlights that similar creatinine-based changes may reflect distinct pathophysiological processes with markedly different prognostic significance. Abbreviations: AKI, acute kidney injury; BNP, B-type natriuretic peptide; BP, blood pressure; GDMT, guideline-directed medical therapy; KIM-1, kidney injury molecule-1; NGAL, neutrophil gelatinase-associated lipocalin; RAASi, renin–angiotensin–aldosterone system inhibitors; SGLT2i, sodium–glucose cotransporter-2 inhibitors; uL-FABP, urinary liver-type fatty acid–binding protein.

Overall, the integration of alternative biomarkers into the assessment of renal function in AHF should be viewed as a complementary strategy rather than a redefinition of AKI or WRF. When interpreted alongside clinical status, congestion markers, and renal function trajectories, these tools may help refine risk stratification, guide therapeutic decisions, and avoid unnecessary de-escalation of HF treatment (Figure 1). However, the absence of standardized thresholds and limited outcome-driven validation currently preclude their routine use as standalone diagnostic criteria in AHF [3,51].

## 7. Is There a Best Definition?

Renal function deterioration in AHF is best conceptualized as a dynamic process rather than a binary event, and the search for a single, universally optimal definition is therefore unlikely to be successful. Rather than reflecting a failure of existing definitions, this likely mirrors the biological and clinical heterogeneity of renal function trajectories observed during AHF treatment [12,32,60].

Creatinine-based thresholds, particularly the widely used definition of WRF as an increase in serum Cr of ≥0.3 mg/dL, have clear practical advantages and remain indispensable in both clinical care and research. Their simplicity allows consistent application across diverse settings and provides a practical initial signal of renal vulnerability. However, their interpretability depends not only on the magnitude of change but also on the choice of baseline Cr and timing of assessment, both of which substantially influence AKI classification and prognostic interpretation [3,30,44,60].

More restrictive definitions—such as higher Cr cut-offs, severe WRF categories, or advanced AKI stages—tend to show stronger and more consistent associations with adverse outcomes. Nevertheless, these approaches identify a smaller subset of patients and may miss clinically relevant but potentially reversible forms of renal dysfunction. Conversely, broader definitions improve sensitivity but at the expense of specificity for true kidney injury. This trade-off highlights that no single Cr-based threshold can simultaneously optimize detection, prognostic accuracy, and clinical interpretability in AHF [29,32].

Importantly, definitions that incorporate clinical modifiers—such as congestion status, timing of renal deterioration, baseline kidney function, and persistence of renal impairment—appear to outperform isolated biochemical criteria. Across studies, renal function changes occurring in the setting of persistent congestion, late in the hospitalization course, or on a background of impaired baseline renal reserve are more consistently associated with adverse outcomes than early, transient changes during effective decongestion [3,27,30,31].

From this perspective, the most clinically meaningful “definition” of AKI or WRF in AHF is not a fixed numerical threshold but a contextual interpretation that integrates biochemical changes with the patient’s hemodynamic status, therapeutic response, and clinical trajectory. Such an approach aligns more closely with the pathophysiology of acute cardiorenal interactions and provides a framework that is better suited to guide management decisions than any standalone definition [3,32,51,61].

## 8. Integrative Interpretation of Renal Function Deterioration in AHF

### 8.1. Interpretation of the Evidence from Clinical Studies

Across the studies reviewed, renal function deterioration in AHF consistently occurs more frequently in patients with pre-existing CKD, higher serum Cr at admission, elevated natriuretic peptide levels, advanced age, and a greater burden of comorbidities. These characteristics define a more advanced cardiorenal phenotype rather than an isolated renal event. In this context, AKI or WRF may act as a marker of reduced renal reserve and systemic vulnerability rather than a direct driver of adverse outcomes [30,33,36,41,42,44].

From a pathophysiological standpoint, CKD reflects a loss of functional nephrons exceeding that expected from normal aging. The commonly used threshold of an eGFR < 60 mL/min/1.73 m^2^ corresponds, in physiological terms, to an approximate loss of more than 50% of functional nephrons, even when total GFR is partially preserved through compensatory increases in single-nephron filtration. As a result, patients with CKD may appear relatively stable at baseline yet possess limited capacity to tolerate hemodynamic stress, congestion, or neurohormonal activation. In such individuals, relatively small changes in serum Cr may reflect exhaustion of renal reserve rather than acute structural injury [45,62]. Importantly, albuminuria represents a complementary dimension of renal vulnerability, reflecting glomerular and tubular injury that may not be captured by creatinine-based estimates of GFR. The coexistence of reduced renal reserve and albuminuria identifies patients at particularly high risk, in whom renal function deterioration during AHF hospitalization likely reflects advanced disease biology rather than treatment-related harm [54,62,63].

Within this framework, worse outcomes observed in patients developing renal function deterioration may therefore be driven largely by the underlying comorbidity burden, hemodynamic instability, and persistent congestion rather than the Cr change itself. This interpretation is supported by studies demonstrating that effective decongestion mitigates the prognostic impact of WRF, whereas persistent congestion remains a dominant determinant of adverse outcomes [27,30].

The modifying role of congestion is further supported by studies integrating clinical signs, biomarkers, and imaging-based assessments. However, because “decongestion” has been defined variably across studies, direct comparison of results and clinical application remains challenging. In this context, a pragmatic multidomain approach may improve interpretive consistency. A minimum assessment may include: (1) improvement in clinical congestion signs (reduction in peripheral edema, jugular venous distension, or pulmonary crackles), (2) downward trajectory of natriuretic peptides, (3) evidence of hemoconcentration (rising hemoglobin, hematocrit, or albumin), and, when feasible (4) ultrasound-based indices such as inferior vena cava dynamics or composite venous congestion scores (e.g., VeXUS). Importantly, discordance between these metrics is common, and no single parameter should be considered definitive in isolation. Concordant improvement across multiple domains provides stronger evidence of effective decongestion and may better contextualize the prognostic significance of Cr changes. These complementary domains are summarized in Table 3 [51,64,65,66,67]. Ultimately, achieving effective volume offloading —rather than avoiding modest Cr fluctuations—appears to be the key determinant of improved prognosis in AHF [6,17,61].

The timing of renal function deterioration adds further complexity. While several studies suggest that early WRF is often functional and related to decongestion, whereas late AKI carries a worse prognosis, the evidence is not uniform [3,34,35,36]. Some data indicate that AKI present at admission or acquired in the community is associated with more pronounced congestion, reduced transglomerular pressure gradient, and clear evidence of tubular injury, likely reflecting prolonged renal ischemia due to venous congestion and/or forward cardiac failure. In contrast, in-hospital AKI developing during therapy often lacks markers of structural tubular injury and appears predominantly hemodynamic in nature. Notably, tubular injury in community-acquired AKI tends to improve during AHF treatment, supporting the dynamic and potentially reversible nature of renal injury in this setting [34,36,68].

Taken together, these findings suggest that neither the presence nor the timing of AKI alone is sufficient to define clinically meaningful renal injury. Instead, renal function deterioration in AHF reflects an interaction between baseline renal vulnerability, congestion severity, hemodynamic status, and therapeutic response.

### 8.2. Clinical Key Points and Their Rationale

From a clinical perspective, several practical principles emerge from the available evidence.

First, awareness of baseline renal function is essential. In the early phase of acute decompensation, serum Cr may be falsely low due to hemodilution. Initiation of decongestive therapy may therefore lead to an apparent “pseudo-WRF,” with Cr rising toward pre-admission baseline values. Such changes should not prompt concern or therapeutic de-escalation in the absence of other signs of renal injury [26,38,69,70].

Second, all methods used to estimate GFR—whether based on serum Cr or Cys C—assume a steady-state condition, which is rarely present during AHF hospitalization. Consequently, dynamic assessment of serum Cr trends, combined with careful evaluation of congestion status and natriuretic peptide trajectories, is more informative than reliance on isolated eGFR values. In cases of significant creatinine rise accompanied by persistent congestion, inadequate diuretic response, or hypotension, reassessment of hemodynamics is warranted. Maintenance of adequate renal perfusion pressure, often approximated by the difference between mean arterial pressure (MAP) and CVP, represents a key therapeutic goal in patients with AHF and renal dysfunction. In clinical practice, a transrenal perfusion pressure greater than approximately 60 mmHg is generally considered a reasonable target to support kidney perfusion, particularly in the setting of congestion or low-output states [71]. In challenging cases, early nephrology involvement with urine sediment examination (e.g., cellular or granular casts) and renal ultrasound (e.g., assessment of size and cortical echogenicity, renal vein doppler) may help distinguish structural kidney injury from functional changes and guide decongestion therapy [3,71].

Third, low muscle mass and cardiac cachexia—frequent in patients with HF, particularly in advanced stages—limit the reliability of creatinine-based assessment and may lead to underestimation of the severity of underlying kidney disease. In this context, Cys C may offer complementary information on renal function and residual renal reserve. Importantly, a marked discrepancy between creatinine- and cystatin C–derived eGFR has been associated with worse clinical outcomes and may reflect heightened neurohormonal activation, frailty, and impaired renal adaptive capacity. Patients exhibiting such discordance often require more cautious decongestion and closer pharmacological monitoring, as they may be more susceptible to treatment intolerance or adverse effects [3,48,49].

In selected cases, adjunctive use of renal tubular injury biomarkers may further aid clinical interpretation. A stable or only mildly fluctuating trajectory of markers such as NGAL, KIM-1, or urinary L-FABP during decongestive therapy is more consistent with functional, hemodynamically mediated changes in renal function and generally supports the continuation of diuretic treatment, whereas significant rises may prompt reassessment of volume status, perfusion, and potential nephrotoxic exposures [3,72,73,74]. Albuminuria may provide complementary information in this setting. In HF cohorts, elevated urinary albumin-to-creatinine ratio (UACR) has been closely associated with markers of volume overload, including NT-proBNP, peripheral edema, and echocardiographic indices of increased right-sided filling pressures. These associations persist independently of GFR and tubular injury markers, suggesting that albuminuria in AHF may reflect venous congestion and systemic endothelial dysfunction rather than intrinsic renal injury alone. Importantly, UACR levels often improve with effective decongestion, and this dynamic response may help distinguish congestion-driven albuminuria from underlying nephropathy [56,57,70].

Fourth, clinicians should recognize that several disease-modifying therapies, including RAASi and SGLT2i, induce predictable reductions in intraglomerular pressure and modest early declines in eGFR. These changes are not indicative of harm but represent mechanisms through which long-term nephron preservation and improved outcomes are achieved [24,30,61].

**Figure 2:**
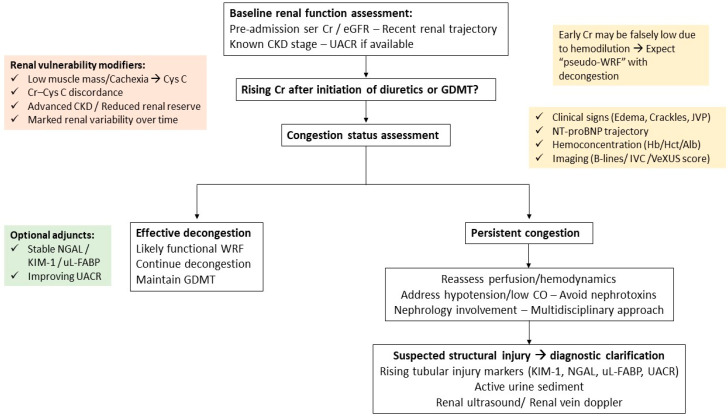
Practical clinical algorithm for contextual interpretation of worsening renal function in acute heart failure. Stepwise clinical algorithm for the evaluation of rising serum creatinine during hospitalization for acute heart failure. The approach emphasizes baseline renal function, assessment of congestion and perfusion status, identification of renal vulnerability modifiers, and selective use of adjunctive biomarkers and imaging. The algorithm distinguishes likely functional worsening renal function from suspected structural kidney injury, supporting individualized decisions regarding decongestive therapy, hemodynamic optimization, and multidisciplinary involvement. Abbreviations: Cr, creatinine; CysC, cystatin C; eGFR, estimated glomerular filtration rate; GDMT, guideline-directed medical therapy; IVC, inferior vena cava; KIM-1, kidney injury molecule-1; NGAL, neutrophil gelatinase-associated lipocalin; NT-proBNP, N-terminal pro–B-type natriuretic peptide; UACR, urinary albumin-to-creatinine ratio; uL-FABP, urinary liver-type fatty acid–binding protein; VeXUS, venous excess ultrasound score.

Fifth, variability in renal function over time may itself identify a high-risk phenotype. Fluctuations in Cr or eGFR across visits likely reflect heightened sensitivity of remaining nephrons to congestion, neurohormonal activation, or hemodynamic stress and are associated with more advanced disease. Stabilization of renal function variability may occur with effective guideline-directed therapy and improved volume control (Figure 2) [71].

## 9. Conclusions and Future Directions

Renal function deterioration during hospitalization for AHF is common and remains a major source of clinical uncertainty. The evidence reviewed suggests that AKI and WRF following initiation of decongestive therapy frequently act as markers of underlying cardiorenal disease severity, rather than direct indicators of isolated structural kidney injury. In many patients, modest increases in serum Cr reflect limited renal reserve, heightened neurohormonal activation, and vulnerability to hemodynamic stress, rather than true tubular damage.

These findings highlight the limitations of interpreting Cr changes in isolation. A more comprehensive patient assessment—integrating clinical evaluation of congestion, serial biomarker trajectories, and imaging-based estimates of volume status and venous congestion—is essential for meaningful interpretation of renal function changes in AHF. Across multiple studies, residual congestion at discharge consistently emerges as a stronger determinant of rehospitalization and mortality than creatinine-defined AKI itself. Consequently, achieving adequate decongestion remains a central therapeutic objective, even in the presence of transient renal function deterioration, provided that renal perfusion is preserved and patients are closely monitored. In this context, appropriate decongestion appears more prognostically relevant than avoidance of modest Cr rises, while excessive diuresis and hypotension should be actively avoided.

Future research should focus on refining the assessment of cardiorenal interactions in AHF, including more accurate and standardized methods to quantify congestion and broader integration of renal biomarkers to better distinguish functional renal changes from structural kidney injury. In this context, emerging stress biomarkers such as NephroCheck^®^ (TIMP-2·IGFBP-7), which reflect early tubular cellular stress preceding overt injury, may provide additional risk stratification. Although primarily validated in high-risk settings, its potential role in advanced HF is increasingly recognized and may support earlier nephroprotective strategies, closer monitoring, and timely optimization of guideline-directed therapy. Ultimately, moving beyond isolated creatinine-based definitions toward context-driven frameworks that reflect cardiorenal disease severity may enable more individualized management and improve outcomes in patients hospitalized with AHF.

## Figures and Tables

**Table 1:** Classification systems for acute kidney injury: RIFLE, AKIN, and KDIGO criteria. Abbreviations: AKIN, Acute Kidney Injury Network; GFR, glomerular filtration rate; KDIGO, Kidney Disease: Improving Global Outcomes; RIFLE, Risk–Injury–Failure–Loss–End-stage; RRT, renal replacement therapy; SCr, serum creatinine [3].

Classification	Stage	Serum Creatinine Criteria	Urine Output Criteria
RIFLE	Risk (R)	Increase in SCr ≥ 1.5× baseline or GFR decrease > 25%	<0.5 mL/kg/h for ≥6 h
	Injury (I)	Increase in SCr ≥ 2.0× baseline or GFR decrease > 50%	<0.5 mL/kg/h for ≥12 h
	Failure (F)	Increase in SCr ≥ 3.0× baseline or SCr ≥ 4.0 mg/dL with acute rise ≥ 0.5 mg/dL or GFR decrease > 75%	<0.3 mL/kg/h for ≥24 h or anuria for ≥12 h
	Loss (L)	Persistent renal failure > 4 weeks	—
	End-stage (E)	End-stage renal disease > 3 months	—
AKIN	Stage 1	Increase in SCr ≥ 0.3 mg/dL or 1.5–1.9× baseline within 48 h	<0.5 mL/kg/h for ≥6 h
	Stage 2	Increase in SCr 2.0–2.9× baseline	<0.5 mL/kg/h for ≥12 h
	Stage 3	Increase in SCr ≥ 3.0× baseline or SCr ≥ 4.0 mg/dL with acute rise ≥ 0.5 mg/dL or initiation of RRT	<0.3 mL/kg/h for ≥24 h or anuria for ≥12 h
KDIGO	Stage 1	Increase in SCr ≥ 0.3 mg/dL within 48 h or 1.5–1.9× baseline within 7 days	<0.5 mL/kg/h for 6–12 h
	Stage 2	Increase in SCr 2.0–2.9× baseline	<0.5 mL/kg/h for ≥12 h
	Stage 3	Increase in SCr ≥ 3.0× baseline or SCr ≥ 4.0 mg/dL or initiation of RRT	<0.3 mL/kg/h for ≥24 h or anuria for ≥12 h

**Table 3:** Pragmatic Multidomain Assessment of Decongestion in Acute Heart Failure. This box summarizes a practical, complementary set of clinical, biochemical, and imaging-based parameters that may be used to assess decongestion during hospitalization for acute heart failure. Because no single metric reliably captures the multidimensional nature of congestion, concordant improvement across domains provides stronger evidence of effective volume offloading and more accurate contextualization of creatinine-based renal function changes. Discordance between parameters is common and should prompt integrative clinical interpretation rather than reliance on isolated findings. Abbreviations: AF, atrial fibrillation; BNP, B-type natriuretic peptide; CVP, central venous pressure; EVEREST, Efficacy of Vasopressin Antagonism in Heart Failure: Outcome Study With Tolvaptan; GDMT, guideline-directed medical therapy; Hb, hemoglobin; Hct, hematocrit; IVC, inferior vena cava; JVD, jugular venous distension; NT-proBNP, N-terminal pro–B-type natriuretic peptide; PCWP, pulmonary capillary wedge pressure; VeXUS, Venous Excess Ultrasound Score.

Domain	Suggested Parameters	Interpretation	Limitations
Clinical examination	Peripheral edema; Dyspnea/ Orthopnea; JVD; Pulmonary crackles; Clinical congestion scores (e.g., EVEREST)	Improvement suggests a reduction in volume overload	Low sensitivity and poor predictive value; Interobserver variability; Reduced reliability in certain patient phenotypes (e.g., obese); Clinical scores may be more prognostic than diagnostic
Natriuretic peptides	BNP/NT-proBNP trajectory	Downward trajectory (≥30%) supports decongestion and more favorable prognosis	Influenced by age, renal dysfunction, AF and initiation or optimization of GDMT
Hemoconcentration	Rise in Hb/Hct; Rise in Albumin	Reflects effective plasma volume contraction; Prognostic value appears strongest when occurring late during hospitalization	Reflects relative plasma volume contraction rather than absolute volume status; Does not quantify true congestion burden; Small changes may be influenced by bleeding, phlebotomy, splenic pooling, or postural variation.
Ultrasound-based markers	IVC diameter + collapsibility; B-lines on lung ultrasound; VeXUS score	Objective estimate of venous congestion; Bedside availability; Minimum training required for basic assessment	IVC not so reliable in positive pressure ventilated patients; More complex scores need more time; B-lines may be present in non-cardiac conditions
Hemodynamics	Reduction in CVP/PCWP	Direct evidence of unloading	Invasive; Not routinely available

## Data Availability

No new data were created or analyzed in this study.

## Abbreviations

The following abbreviations are used in this manuscript:

ACS	Acute Coronary Syndrome
ACRS	Acute cardiorenal syndrome
ADHF	Acute decompensated heart failure
AF	Atrial Fibrillation
AHF	Acute heart failure
AKI	Acute kidney injury
AKIN	Acute Kidney Injury Network
BNP	B-type natriuretic peptide
CA-AKI	Community-acquired acute kidney injury
CKD	Chronic kidney disease
Cr	Creatinine
CVP	Central venous pressure
Cys C	Cystatin C
eGFR	Estimated glomerular filtration rate
EVEREST	Efficacy of Vasopressin Antagonism in Heart Failure: Outcome Study With Tolvaptan
GDMT	Guideline-directed medical therapy
Hb	Hemoglobin
Hct	Hematocrit
HF	Heart Failure
HFpEF	Heart Failure with preserved ejection fraction
ICU	Intensive Care Unit
IGFBP-7	Insulin-like growth factor–binding protein 7
KDIGO	Kidney Disease: Improving Global Outcomes
KIM-1	Kidney injury molecule-1
L-FABP	Liver-type fatty acid–binding protein
MAP	Mean Arterial Pressure
MeSH	Medical Subject Headings
NGAL	Neutrophil gelatinase–associated lipocalin
NT-proBNP	N-terminal pro B-type natriuretic peptide
PCWP	Pulmonary capillary wedge pressure
RAAS	Renin–Angiotensin–Aldosterone system
RAASi	Renin–Angiotensin–Aldosterone system inhibitors
RCT	Randomized controlled trial
RIFLE	Risk–Injury–Failure–Loss–End-stage
RRT	Renal replacement therapy
SCr	Serum Creatinine
SGLT2i	Sodium–glucose cotransporter-2 inhibitors
TIMP-2	Tissue inhibitor of metalloproteinases-2
VeXUS	Venous excess ultrasound score
UACR	Urinary Albumin—to—Creatinine Ratio
uL-FABP	Urinary Liver-type fatty acid–binding protein
WRF	Worsening renal function
